# The dynamics of a Mediterranean coralligenous sponge assemblage at decennial and millennial temporal scales

**DOI:** 10.1371/journal.pone.0177945

**Published:** 2017-05-22

**Authors:** Marco Bertolino, Gabriele Costa, Mirko Carella, Riccardo Cattaneo-Vietti, Carlo Cerrano, Maurizio Pansini, Gianluca Quarta, Lucio Calcagnile, Giorgio Bavestrello

**Affiliations:** 1 DiSTAV, Università di Genova, Corso Europa, Genova, Italy; 2 CEAB Carrer accés Cala Sant Francesc, Blanes, Spain; 3 DiSVA, Università Politecnica delle Marche, Via Brecce Bianche, Ancona, Italy; 4 CEDAD, Università del Salento, Via per Arnesano, Lecce, Italy; University of Padova, ITALY

## Abstract

This paper concerns the changes occurred over both decennial and millennial spans of time in a sponge assemblage present in coralligenous biogenic build-ups growing at 15 m depth in the Ligurian Sea (Western Mediterranean). The comparison of the sponge diversity after a time interval of about 40 years (1973–2014) showed a significant reduction in species richness (about 45%). This decrease affected mainly the massive/erect sponges, and in particular the subclass Keratosa, with a species loss of 67%, while the encrusting and cavity dwelling sponges lost the 36% and 50%, respectively. The boring sponges lost only one species (25%). This changing pattern suggested that the inner habitat of the bioconstructions was less affected by the variations of the environmental conditions or by the human pressures which, on the contrary, strongly affected the species living on the surface of the biogenic build-ups. Five cores extracted from the bioherms, dating back to 3500 YBP, allowed to analyse the siliceous spicules remained trapped in them in order to obtain taxonomic information. Changes at generic level in diversity and abundance were observed at 500/250-years intervals, ranging between 19 and 33 genera. The number of genera showed a sharp decrease since 3500–3000 to 3000–2500 YBP. After this period, the genera regularly increased until 1500–1250 YBP, from when they progressively decreased until 1000–500 YBP. Tentatively, these changes could be related to the different climatic periods that followed one another in the Mediterranean area within the considered time span. The recent depletion in sponge richness recorded in the Ligurian coralligenous can be considered relevant. In fact, the analysis of the spicules indicated that the sponges living in these coralligenous habitats remained enough stable during 3000 years, but could have lost a significant part of their biodiversity in the last decades, coinciding with a series of warming episodes.

## Introduction

The Mediterranean littoral hard bottoms are often characterized by coralligenous communities that develop, with different growth patterns, depending on sedimentation rates, water transparency, substrate mineralogy and morphology [1, 2]. Coralligenous substrata are biogenic build-ups, mainly due to the carbonate deposition of several encrusting coralline algae (*Lithophyllum*, *Lithothamnion*, *Mesophyllum* and *Peyssonnelia*) growing in dim light conditions [3, 4, 5] and, in lesser extent, to the calcareous skeletons of some benthic animals [6, 7, 8].

These bioconstructions form a complex mosaic of micro-habitats, with crevices and cavities, produced by the irregular growth of algal talli and by the destructive action of some boring organisms, such as sponges and bivalves [2]. The age of these build-ups, estimated by ^14^C, can also go back to 8000 YBP [5, 9, 10, 11], when the Mediterranean Sea level was lower than now, although, after 6000 YBP, the deepest build-ups stopped developing and the carbonate deposition appeared negligible [5].

These build-ups may develop both on vertical cliffs, where they produce a series of overhanging ridges with the growth axis perpendicular to the rocky wall [2], but also on flat rocky bottoms, often forming columnar structures diffused in shallow waters, between 10 and 30 m depth [8, 12]. These structures are younger than the coralligenous settled on the vertical cliffs because they started to grow when the sea level increased. They show a slow growth rate (0.2–4 mm/year), reach a thickness comprised between 0.5 and 3.4 m, and are often characterized by cavernous structures [8, 13].

Sponges are the most representative animal group of these habitats, with more than 300 recorded species, living in different zones of the bioconstructions and showing different growth patterns. Massive/erect species live on the surface of the build-ups, others encrust their surface or fill crevices and holes and, finally, boring species actively excavate the carbonate structure [14]. The different sponge categories contribute, according to their different habitus, to the strengthening, stabilization or erosion of the bio-carbonate structures [15].

Recently, Bertolino *et al*. [11] pointed out a method, based on the study of free spicules embedded in the sediment filling the inner holes of coralligenous accretions, to identify species living in the past on and inside these structures. The spicule analysis, coupled with the age estimation of the coralligenous containing the spicular remains, allowed the reconstruction of the sponge fauna of the bioconstructions along their entire life span.

**Fig 1:**
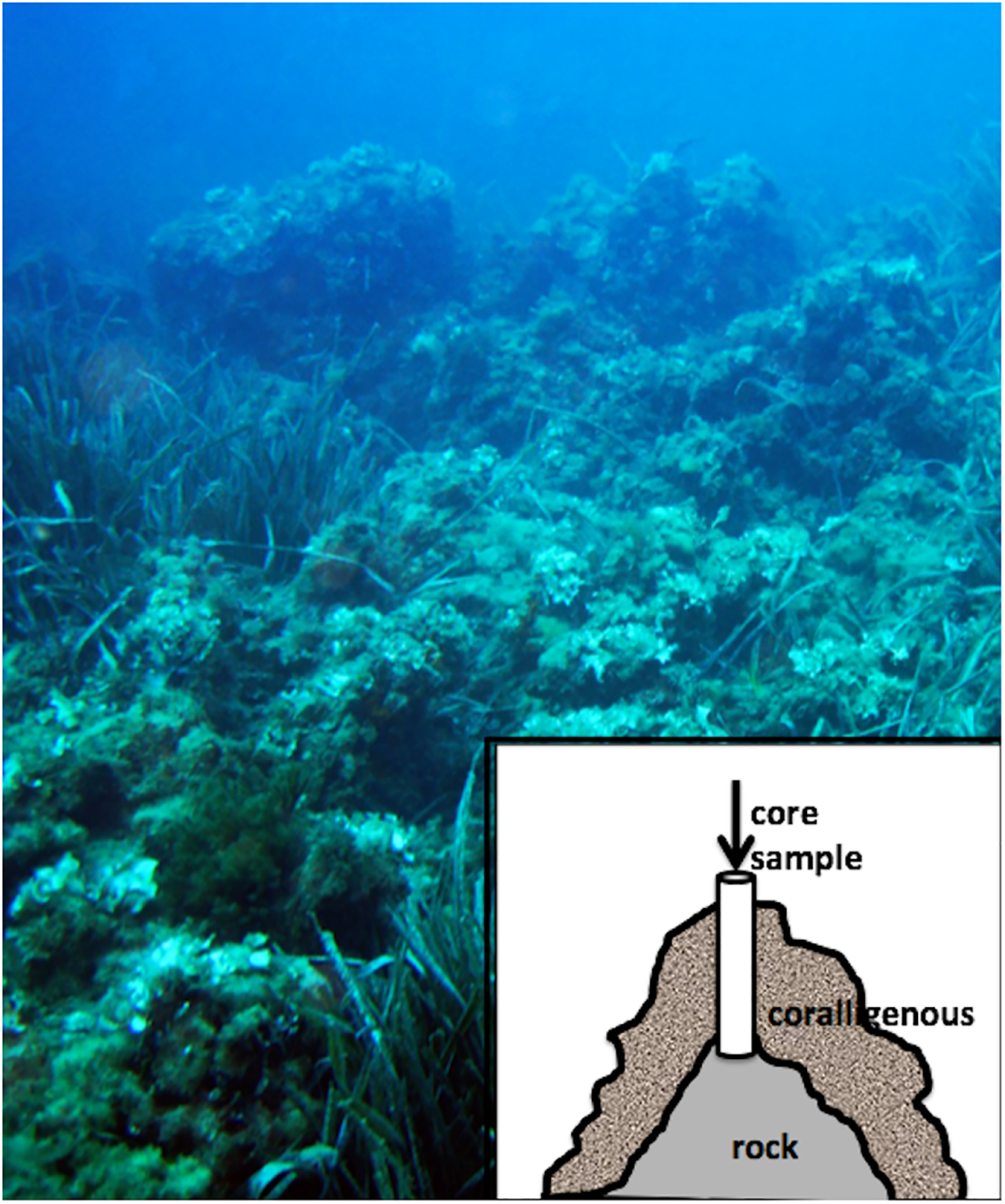
A view of the studied coralligenous of Bogliasco (Ligurian Sea) mixed with patches of the seagrass *Posidonia oceanica*. The drawing shows a core sample conduced on a build-up and reaching the basal rock.

The present study was dedicated to the description of the sponge fauna living on the coralligenous build-ups (Fig 1) arising from a flatted bottom 15 m depth, off the Bogliasco marina (Ligurian Sea, Western Mediterranean Sea), at two different temporal scales. At a decennial span of time, we have compared the actual sponge fauna (2014) with the records obtained from the same habitat 40 years before by Pansini and Pronzato [16]. During this span of time, in the '70 and '80s years of the past century, the outcrops were subject to the destructive date-mussel fishery, at last forbidden by the Italian law in 1998. Moreover, thanks to the study of five cores of the bioherms going back 3500 YBP, the dynamics of the sponge fauna along the last part of the Holocene were investigated, analysing the siliceous spicules remained trapped inside the bioconstruction, when the sponge dies.

## Material and methods

The studied sponge fauna was present in the coralligenous build-ups, arising on a flat bottom 15 m deep, on a belt 70–90 m wide, 300 m off the Bogliasco marina (Ligurian Sea, Western Mediterranean) (44,374418° N, 9,06734° E) (Fig 1). Sampling on the build-ups was performed by SCUBA diving, during summer 2014. Fifteen standard areas of 400 cm^2^ (20 x 20 cm) were completely scraped using hammer and chisel to a depth of 3 cm in the aim of collecting also the sponges growing inside the bioherms. The collected sponges were sorted and identified at species level in laboratory. The sponge abundance was estimated as the per cent presence of each species on the total scraped areas. The obtained data were compared with those recorded during a survey conducted in 1973 with the same methods and in the same area [16].

Moreover, to study the structure of the sponge assemblage during the entire life of the build-ups, five core samples were obtained by a pneumatic corer operated by professional divers from five different build-ups (Fig 1). All the samples were conducted from top to bottom of the accretions, in order to reach the basal rock. The core samples had a length ranging from 9 cm to 45 cm.

According to the method proposed by Bertolino *et al*. [11] and improved in this work, the core samples were divided in 3 cm thick portions by a stone-saw. Each portion was placed in hydrogen peroxide (240 vol) that was changed three times at intervals of 24h. This method released all the sediment entrapped in the cavities of the bioherm. The pieces of biocarbonates were removed and dried, while the obtained sediment was treated with boiling nitric acid to eliminate the carbonate fraction, rinsed twice in distilled water and twice in alcohol 95%, and dried. For each sample of the sediment, three replicates of 10 mg each were mounted on a microscope slide. Microscopic analysis of the spicules contained in the sediment was done to investigate the sponge species living in ancient times. Whenever possible, the embedded spicules were attributed to a genus. Finally each spicule type was counted.

After the sediment extraction, each portion of the bioherm was ^14^C dated by Accelerator Mass Spectrometry (AMS) at the Center of Dating and Diagnostic (CEDAD) of the University of Salento [17] (Table 1).

**Table 1:** Calibrated radiocarbon ages of the different layers of each core sample. Uncertainty refers to one standard deviation confidence level. Present assumed as 1950AD.

Distance from the basal rock	Core samples				
	B1	B2	B3	B4	B5
cm	Years Before Present (YBP)				
0–3	1024 ± 65	1019 ± 65	2833 ± 65	1367 ± 60	1974 ± 65
3–6	2082 ± 75	1483 ± 65	2806 ± 55	517 ± 38	3442 ± 55
6–9	1422 ± 60	1884 ± 60	3423 ± 60	948 ± 55	2422 ± 80
9–12	1298 ± 45	1492 ± 65		1757 ± 65	871 ± 55
12–15	1826 ± 65	2240 ± 65		1877 ± 60	3104 ± 75
15–18	1356 ± 55	1671 ± 70		1698 ± 70	
18–21	1970 ± 65	1040 ± 65		1408 ± 65	
21–24	560 ± 45	1318 ± 45		1311 ± 45	
24–27		1247 ± 45		620 ± 55	
27–30		801 ± 60			
30–33		1214 ± 50			
33–36		1363 ± 60			
36–39		957 ± 55			
39–42		1374 ± 60			
42–45		1010 ± 60			

Samples were converted to carbon dioxide by acid hydrolysis (H_3_PO_4_) and the extracted CO_2_ was converted to graphite after cryogenic purification [18]. Conventional radiocarbon age were then calculated from the ^14^C/^12^C isotopic ratios measured with the AMS system after correcting for isotopic fractionation and chemical processing and machine background. Conventional radiocarbon ages were then calibrated in calendar years by using the MARINE13 [19] curve and a ΔR = 58±15 as average value for the Mediterranean Sea [20]. Calibrated radiocarbon ages were expressed as years cal BP and used in the following chronological discussion and interpretations.

Since the age determination of each portion of bioherm appeared inconsistent with a temporal growth in all the build-ups, we have considered each portion independently, assuming that the spicules present may have approximately the same age as the surrounding bio-deposed carbonates. This assumption is coherent with the fact that spicules of boring and cavity dwelling sponges remain *in situ* when they die. On the other hand, we hypothesise that a large part of the spicular remains of massive/erected or encrusting species fall down and accumulate in the coralligenous crevices, although their incorporation could be partially biased by local hydrodynamic conditions. By this way, we have obtained the trend of sponge diversity, evaluated as number of recorded genera, and the trend of the amount of the spicules entrapped in the build-ups along a span of time ranging from 3500 to 600 YBP and subdivided in intervals of 250/500 years each.

## Ethics statement

We confirm that the coralligenous community of Bogliasco is not included in any Marine Protected Area, that no specific permissions are required to work in this location and on this material and that the field studies did not involve endangered or protected species.

## Results

The studied coralligenous build-ups, present off the Bogliasco coast on a rocky bottom at 15 m depth, were irregular in shape, about 40–70 cm high, rich in crevices and holes. They were covered by a dense community of photophylous algae (mainly *Padina pavonica*, *Acetabularia acetabulum*, *Codium* spp., *Dictyota dichotoma*, *Dyctiopteris* spp.) while patches of the sea grass *Posidonia oceanica* and sandy pouches were dispersed among the coralligenous accretions (Fig 1).

**Fig 2:**
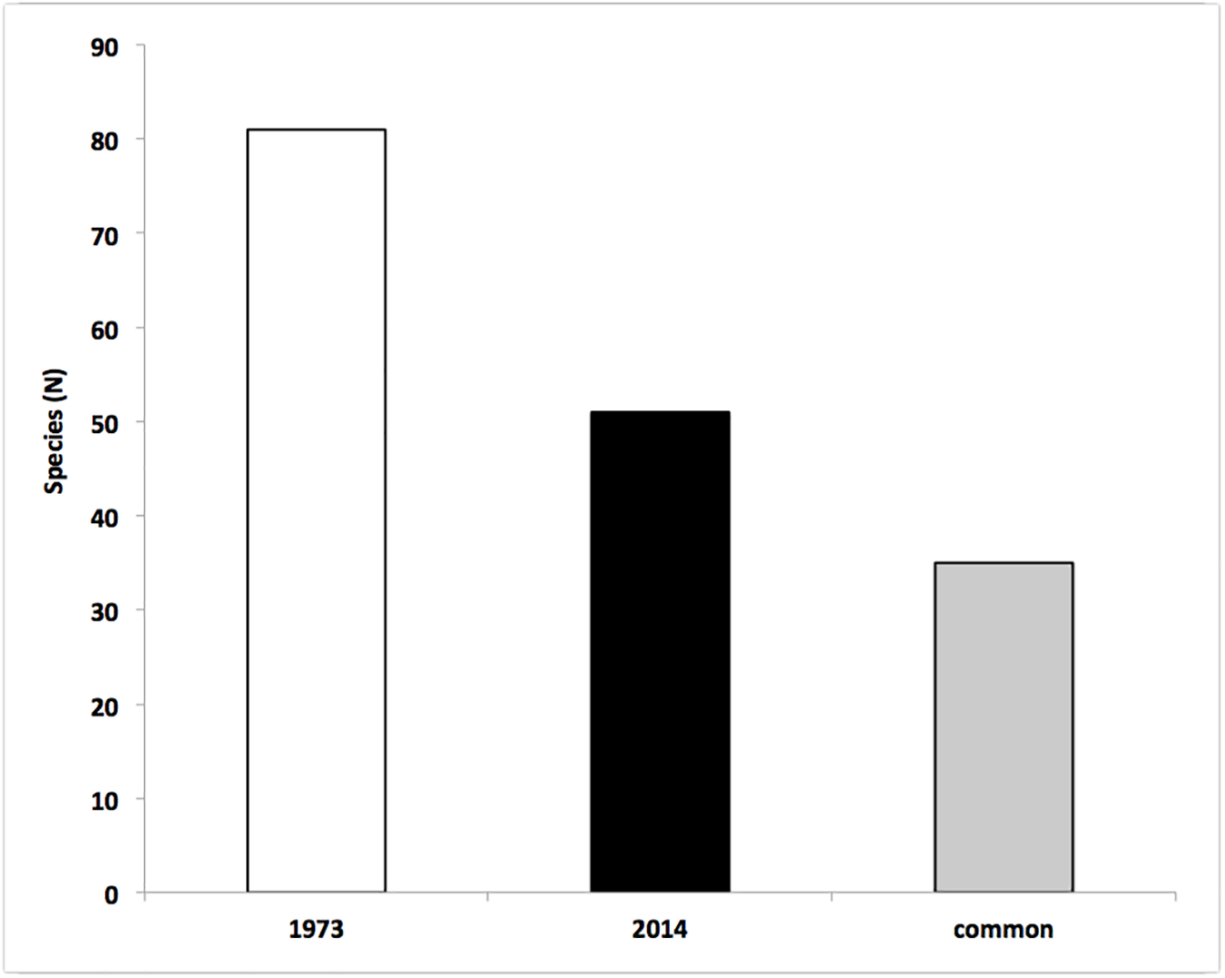
Sponge species number recorded in the Bogliasco coralligenous assemblage during the samplings of 1973 and 2014. The grey bar indicates the species number shared by the two surveys.

**Fig 3:**
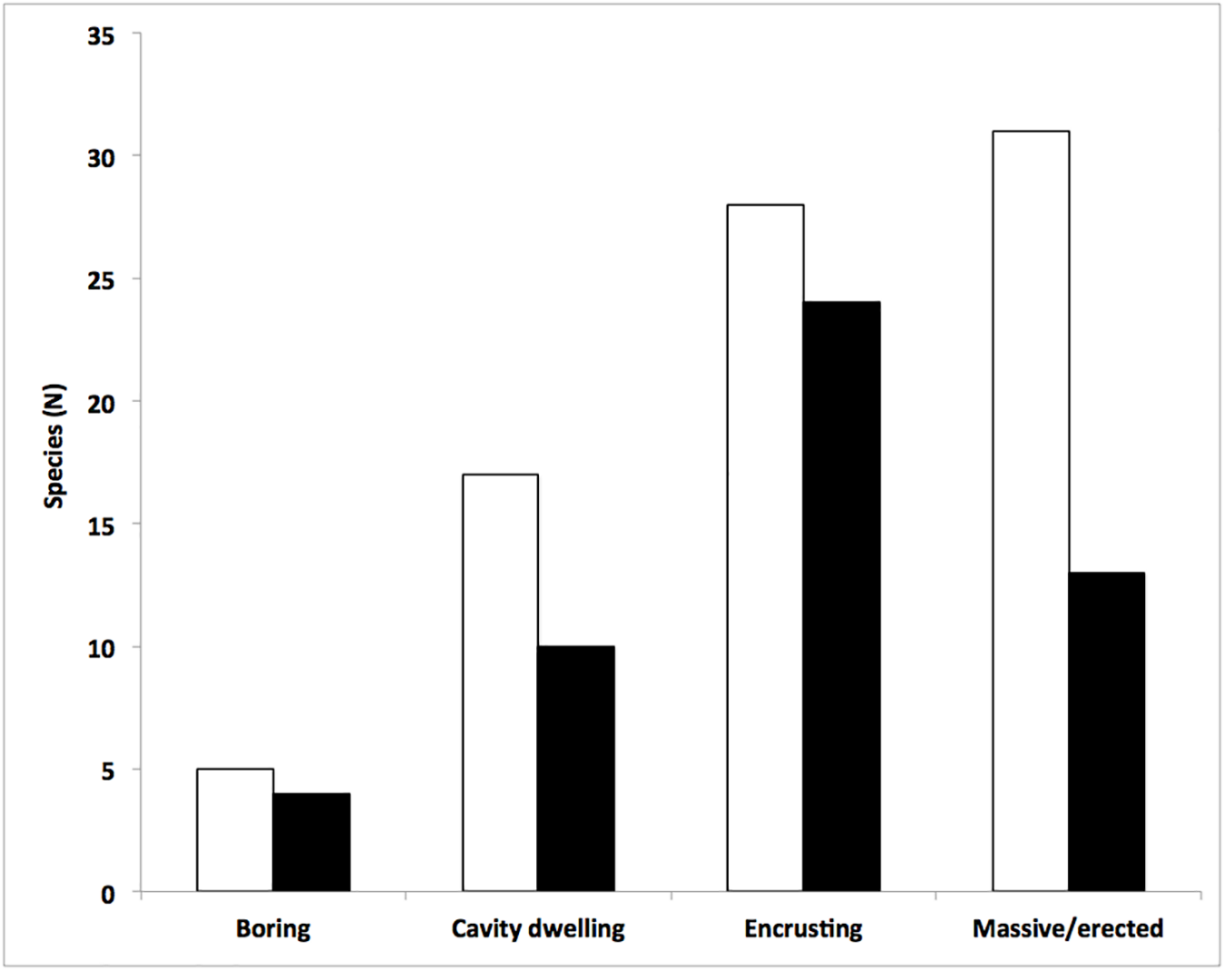
Sponge species number recorded in the Bogliasco coralligenous assemblage during the samplings of 1973 (white bars) and 2014 (black bars), according to the sponge different growth patterns. The main reduction was recorded within the massive/erect sponges.

The sponge fauna settled on these bioherms sampled in 2014 was composed by 51 species (Table 2, S1 Table), with variable abundances among different accretions. The average number of species per scraped area was 8.6 ± 0.97 with maximal of 15 and minimal of 4 species. Considering the complex of the recorded species some differences were observed according to the different kinds of growth pattern (massive/erect, encrusting, cavity dwelling, and boring) (Figs 2 and 3). The most numerous species belonged to the category of the encrusting sponges (24 species), followed by massive/erect (13 species), cavity dwelling (10 species) and boring (4 species). The cavity dwelling *Jaspis johnstonii* and *Dercitus* (*Stoeba*) *plicatus*, together with the encrusting *Crambe crambe*, were the most frequent, present in almost 60% of the samples. Other common species, present in more than 40% of the samples, were *Phorbas tenacior* (encrusting), *Chondrosia reniformis* (massive) and *Jaspis incrustans* (cavity dwelling).

**Table 2:** Percent abundance and growth pattern of the sponge species recorded during the two surveys. The species in bold were shared by the two surveys.

Species	Growth pattern	Pansini & Pronzato (1973)	Present paper
***Agelas oroides* (Schmidt, 1864)**	ME	0–10	20
*Hymerhabdia oxytrunca* Topsent, 1904	Ec	21–40	0
***Axinella damicornis* (Esper,1794)**	ME	0–10	13,3
*Axinella polypoides* Schmidt, 1862	ME	P	0
*Axinella* sp.	ME	0–10	0
***Eurypon cinctum* Sarà, 1960**	Ec	P	6,6
*Eurypon clavatum* (Bowerbank, 1866)	Ec	0–10	0
*Eurypon* cf. *clavatum* (Bowerbank, 1866)	Ec	0	6,6
***Eurypon major* Sarà & Siribelli,1960**	Ec	P	13,3
***Eurypon vesciculare* Sarà & Siribelli,1960**	Ec	60–80	13,3
*Eurypon viride* (Topsent, 1889)	Ec	11–20	0
***Eurypon* sp.**	Ec	0–10	6,6
*Raspaciona aculeata* (Johnston, 1842)	Ec	21–40	0
***Bubaris vermiculata* (Bowerbank, 1866)**	Ec	60–80	6,67
*Desmanthus incrustans* (Topsent, 1889)	Ec	0–10	0
***Acanthella acuta* Schmidt, 1862**	ME	P	20
***Dictyonella incisa* (Schmidt,1880)**	ME	P	13,3
*Dictyonella pelligera* (Schmidt, 1862)	Ec	0–10	0
***Cliona celata* Grant, 1826**	Br	0–10	6,66
***Cliona janitrix* Topsent, 1932**	Br	21–40	6,66
*Cliona lobata* Hancock, 1849	Br	0–10	0
*Cliona schmidtii* (Ridley, 1881)	Br	0	13,3
***Cliona viridis* Schmidt, 1862**	Br	21–40	26,6
*Cliona* sp.	Br	0–10	0
***Spirastrella cunctatrix* Schmidt, 1868**	Ec	11–20	13,3
*Diplastrella bistellata* (Schmidt, 1862)	Ec	21–40	0
*Haliclona* (*Gellius*) *angulata* (Bowerbank, 1866)	Cd	11–20	0
*Haliclona* (*Gellius*) *dubia* (Babic, 1922)	Ec	P	0
*Haliclona* (*Gellius*) lacazei (Topsent, 1893)	Ec	0	13,3
***Haliclona* (*Gellius*) sp.**	Ec	0–10	6,6
***Haliclona* (*Halichoclona*) *fulva* (Topsent, 1893)**	Ec	0–10	6,6
*Haliclona* (*Reniera*) cf. *mediterranea* Griessinger, 1971	Ec	0	13,3
*Haliclona* (*Reniera*) *cinerea* (Grant, 1826)	Ec	21–40	0
*Haliclona* (*Reniera*) *cratera* (Schmidt, 1862)	ME	0–10	0
***Haliclona* (*Reniera*) sp.**	Ec	11–20	20
***Haliclona* (*Soestella*) *valliculata* (Griessinger, 1971)**	Ec	11–20	13,3
***Petrosia* (*Petrosia*) *ficiformis* (Poiret, 1789)**	ME	P	26,6
*Oceanapia* sp.	ME	0	6,6
*Acarnus tortilis* Topsent, 1892	Cd	0–10	0
*Batzella inops* (Topsent, 1891)	Ec	0	6,67
***Crambe crambe* (Schmidt,1862)**	Ec	21–40	73
*Hymedesmia* (*Hymedesmia*) *baculifera* (Topsent, 1901)	Ec	0	6,6
*Hymedesmia* (*Hymedesmia*) cf. *gracilisigma* Topsent, 1928	Ec	0	6,6
*Hymedesmia* (*Hymedesmia*) sp.	Ec	0	6,6
*Phorbas fictitius* Bowerbank, 1866	Ec	0	13,3
***Phorbas tenacior* (Topsent,1925)**	Ec	11–20	40
***Mycale* (*Mycale*) *massa* (Schmidt, 1862)**	ME	0–10	13,3
*Myxilla* (*Myxilla*) *rosacea* (Lieberkühn, 1859)	Ec	0	6,6
***Polymastia inflata* Cabioch, 1968**	ME	11–20	6,6
***Halichondria* (*Halichondria*) *bowerbanki* Burton, 1930**	ME	21–40	6,6
*Halichondria* (*Halichondria*) *contorta* (Sarà, 1961)	Cd	21–40	0
***Halichondria* (*Halichondria*) *genitrix* (Schmidt, 1870)**	Cd	11–20	20
*Halichondria* (*Halichondria*) *semitubulosa* Lieberkühn, 1859	ME	P	0
***Halichondria* sp.**	Cd	P	20
*Hymeniacidon perlevis* (Montagu, 1818)	Ec	0	6,6
***Aaptos aaptos* (Schmidt, 1864)**	Cd	21–40	27
*Protosuberites epiphytum* (Lamarck, 1815)	Ec	0–10	0
*Suberites carnosus* (Johnston, 1842)	ME	0–10	0
***Terpios gelatinosa* (Bowerbank, 1866)**	Ec	11–20	6,6
*Tethya aurantium* (Pallas, 1766)	ME	0–10	0
*Tethya citrina* Sarà & Melone, 1965	ME	21–40	0
*Timea fasciata* Topsent, 1934	Ec	21–40	0
*Timea irregularis* Sarà & Siribelli, 1960	Ec	0–10	0
*Timea stellata* (Bowerbank, 1866)	Cd	41–60	0
***Dercitus* (*Stoeba*) *plicatus* (Schmidt, 1868)**	Cd	21–40	73,3
*Jaspis incrustans* (Topsent, 1890)	Cd	0	33,3
***Jaspis johnstonii* (Schmidt,1862)**	Cd	41–60	66,6
*Stelletta dorsigera* Schmidt, 1864	ME	0–10	0
*Stelletta lactea* Carter, 1871	Cd	0	6,6
***Erylus discophorus* (Schmidt, 1862)**	Cd	21–40	20
*Erylus euastrum* (Schmidt, 1868)	Cd	21–40	0
*Geodia cydonium* Schmidt,1862	Cd	0–10	0
*Penares helleri* (Schmidt, 1864)	Cd	21–40	0
*Caminella intuta* (Topsent, 1892)	ME	P	0
*Pachastrella monilifera* Schmidt, 1868	Cd	0–10	0
*Triptolemma simplex* (Sarà, 1959)	Cd	41–60	0
*Trachycladus minax* (Topsent, 1888)	ME	0–10	0
*Spongionella pulchella* (Sowerby, 1804)	ME	11–20	0
*Dysidea fragilis* (Montagu, 1818)	ME	0–10	0
*Dysidea* sp.	ME	11–20	0
*Pleraplysilla spinifera* (Schulze, 1879)	Ec	0–10	0
*Ircinia dendroides* (Schmidt, 1862)	ME	0–10	0
*Ircinia variabilis* (Schmidt, 1862)	ME	0	6,6
*Sarcotragus fasciculatus* (Pallas, 1766)	ME	P	0
***Sarcotragus spinosulus* Schmidt, 1862**	ME	P	13,3
*Spongia (Spongia) nitens* (Schmidt, 1862)	ME	P	0
*Spongia* (*Spongia*) *officinalis* Linnaeus, 1759	ME	0–10	0
***Spongia* (*Spongia*) *virgultosa* (Schmidt, 1868)**	Cd	41–60	20
*Spongia* (*Spongia*) *zimocca* Schmidt, 1862	ME	0–10	0
*Cacospongia mollior* Schmidt, 1862	ME	P	0
***Fasciospongia cavernosa* (Schmidt, 1862)**	Cd	P	6,6
*Scalarispongia scalaris* (Schmidt, 1862)	ME	0–10	0
***Chondrosia reniformis* Nardo,1847**	ME	0–10	33,3
*Corticium candelabrum* Schmidt, 1862	ME	0–10	0
*Plakina dilopha Schulze*, *1880*	Ec	P	0
***Plakina trilopha* Schulze, 1880**	Ec	P	13,3
*Oscarella* sp.	ME	0	13,3
Boring species (Br)		5	4
Cavity dwelling species (Cd)		17	10
Encrusting species (Ec)		28	24
Massive/erect species (ME)		31	13
Total recorded species		81	51
Total species in common: 35			

Pansini and Pronzato [16], studying the same sponge assemblage in the same locality (Fig 2), recorded 81 species (Table 2). At that time, according to the growth patterns, the massive/erect (31 species) and the encrusting (28 species) were the most representative (Fig 3). The most abundant sponges were *Eurypon vescicularis* and *Bubaris vermiculata* present at least in 60% of the samples. *Spongia* (*Spongia*) *virgultosa*, *Jaspis johnstonii*, *Triptolemma simplex* and *Timea stellata* were recorded in more than 40% of the examined samples.

Comparing the results of the two surveys separated by a 40 years time span, a strong loss of sponge diversity was observed. On a total of 97 recorded species in both the study periods, only 35 species (36%) were in common, while 16 species were recorded as new entries in 2014 and 46 species, observed in 1973, were lost (Fig 2). This decrease was not homogenously distributed among the sponge growth patterns: the massive/erect species experienced the major loss (21 lost species, 68%), while the encrusting and cavity dwelling sponges lost 14 (50%) and 9 species (52%) respectively, while the boring ones lost only 1 species (25%) (Fig 3). A similar pattern was observed also at generic level with a loss of 20% of the genera recorded in 1973. The only boring genus (*Cliona*) was shared by both surveys; the number of cavity dwelling and encrusting genera was very similar, while the massive/erected genera lost 40%.

From a taxonomic point of view, the most impressive reduction involved the subclass Keratosa that dropped from 14 species in 1973 to only 4 species in 2014. Also the species belonging to the sub-order Astrophorina were strongly reduced, shifting from 9 to 5.

Other species, very abundant in 1973, were absent in 2014: for example, *Triptolemma simplex* and *Timea stellata* were not more recorded, as well as the other two species of the genus *Timea* (*T*. *fasciata* and *T*. *irregularis*). The most frequently recorded species in 1973, *Eurypon vescicularis* and *Bubaris vermiculata*, although still present in 2014, showed a reduction in their abundance. On the contrary, the genus *Hymedesmia*, not found in 1973, was now recorded with at least three species.

The study of the core samples indicated that the bioherms were always based on portions of rock emerging from the substratum. The thickness of these build-ups ranged, in the five considered core samples, from 9 to 45 cm, and their ages, estimated by ^14^C analysis, covered a span of time comprised between 600 and 3500 YBP (Table 1).

The age determination of all the examined cores indicated a non-coherent sequence of the age of the layers with a linear temporal growth, showing older layers overlapping the younger ones.

**Fig 4:**
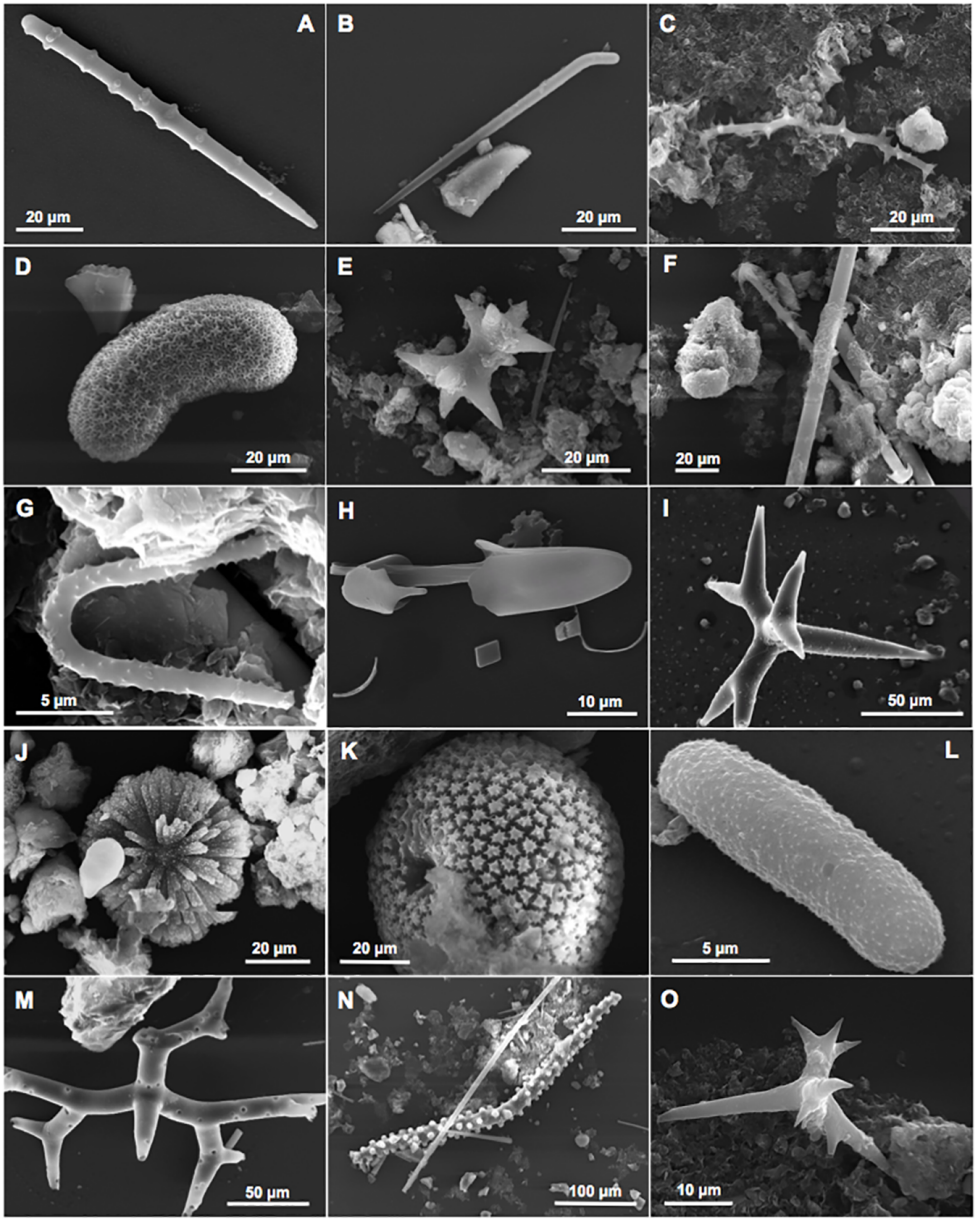
Examples of spicules embedded in the sediment inside the coralligenous crevices. A, achantostyle of *Agelas*; B, rhabdostyle of *Rhabderemia*; C, spiraster of *Cliona*; D, selenaster of *Placospongia*; E, diplaster of *Diplastrella*; F, cladotylote of *Acarnus*; G, forceps of *Forcepia*; H, anisochela of *Mycale*; I, dichotriene of *Dercitus*; J, sterraster of *Erylus*; K, sterraster of *Geodia*; L, microstrongyle of *Pachastrella*; M, dichomesotriene of *Triptolemma*; N, tubercolate oxea of *Alectona*; O, dilophose calthrop of *Plakina*.

A gross reconstruction of the Bogliasco coralligenous sponge assemblages over a span of time of about 3000 years was possible thanks to the taxonomic analysis of the siliceous spicules embedded in the sediment contained into the cavities of these bioherms (Fig 4). Forty genera in total were recognisable in the assemblage: 4 of them were of boring sponges, 14 genera of cavity dwelling and encrusting species, and 8 genera of massive/erect sponges (Table 3, S2, S3, S4, S5 and S6 Tables).

**Table 3:** List of the genera identified on the basis of spicular remains recorded in the layers belonging to the considered spans of time. Genera in bold were also recorded in the recent surveys.

Recorded genera	Growth pattern	Considered spans of time (YBP)						
		500–1000	1000–1250	1250–1500	1500–2000	2000–2500	2500–3000	3000–3500
***Agelas***	Ms		X	X				
*Alveospongia*	Ms	X	X	X	X	X		X
***Eurypon***	Ec	X	X	X	X	X	X	X
*Rhabderemia*	Ms	X	X	X	X	X	X	X
***Bubaris***	Ec				X			
***Acanthella***	Ms	X	X	X	X	X	X	
***Cliona***	Br	X	X	X	X	X	X	X
*Dotona*	Br	X	X	X	X	X	X	X
*Spiroxya*	Br	X	X	X	X	X	X	X
*Placospongia*	Ec	X		X	X		X	X
***Diplastrella***	Ec	X	X	X	X	X	X	X
*Dendroxea*	Cd		X	X				
***Petrosia***	Ms	X	X	X	X	X		
***Acarnus***	Cd	X	X	X	X	X	X	X
***Batzella***	Ec		X		X			
*Forcepia*	Ec	X	X	X				
*Crella*	Ec	X	X	X	X	X		X
*Clathria*	Ec				X	X		
*Antho*	Ec	X	X	X	X	X	X	X
***Mycale***	Ec	X						
***Myxilla***	Ec					X		
***Aaptos***	Cd	X	X	X	X	X	X	X
***Protosuberites***	Ec	X	X	X	X	X	X	X
***Tethya***	Ms	X	X	X	X	X		X
***Timea***	Cd	X	X	X	X	X	X	X
***Dercitus***	Cd	X	X	X	X	X	X	X
***Jaspis***	Cd	X	X	X	X	X	X	X
***Stelletta***	Cd	X	X	X	X	X		X
***Erylus***	Cd	X	X	X	X	X	X	X
***Geodia***	Cd	X	X	X	X	X	X	X
***Penares***	Cd	X	X	X	X			
***Pachastrella***	Cd		X	X				
***Triptolemma***	Cd			X	X			
*Alectona*	Br			X				X
*Thoosa*	Cd	X		X	X	X	X	X
*Samus*	Cd	X	X	X	X	X		X
*Chondrilla*	Ms	X	X	X	X			X
***Corticium***	Ms		X					
***Plakina***	Ec	X	X	X	X	X	X	X
*Plakortis*	Ec		X			X		
Boring genera (Br)		3	3	4	3	3	3	4
Cavity dwelling genera (Cd)		11	12	14	12	10	8	10
Encrusting genera (Ec)		8	7	7	9	7	5	6
Massive/erect genera (ME)		6	8	7	6	5	2	4
Total recorded genera		29	32	33	31	27	19	25

**Fig 5:**
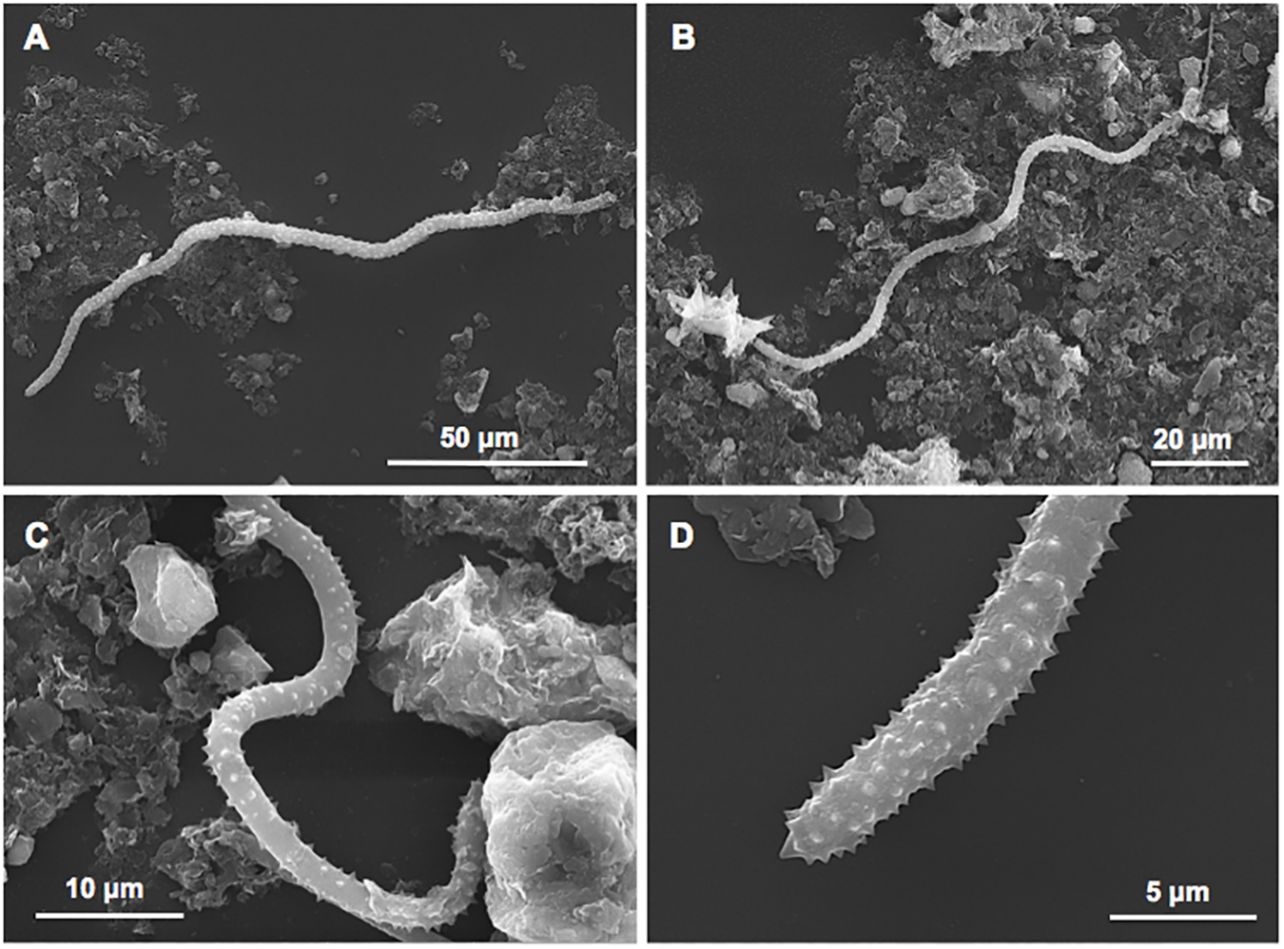
*Alveospongia* sp. A, B two different examples of sinuous acanthomicrostrongyles typical of the genus; C, detail of the microspiny surface of acanthomicrostrongyles; D, magnification of the acanthomicrostrongyle tip.

Twenty-four genera recorded in the ancient-assemblages (60%) were observed again in the recent surveys (1973, 2014). However, among the four recorded genera of boring sponges, only *Cliona* was still present in the recent surveys, while *Spiroxya*, *Dotona* and *Alectona* were no more detected. Seventy-one, 58 and 57% of cavity dwelling, encrusting and massive/erected species respectively were in common with the recent sponge fauna of the build-ups. In particular, among the massive/erect genera, we have recorded the genus *Alveospongia* (fam. Heteroxyidae), determined on the basis of spiny microrhabdose microscleres (Fig 5). This genus, recently described in shallow-waters off Canavieiras (Bahia, Brazil) [21] was, until now, unknown in the Mediterranean Sea.

**Fig 6:**
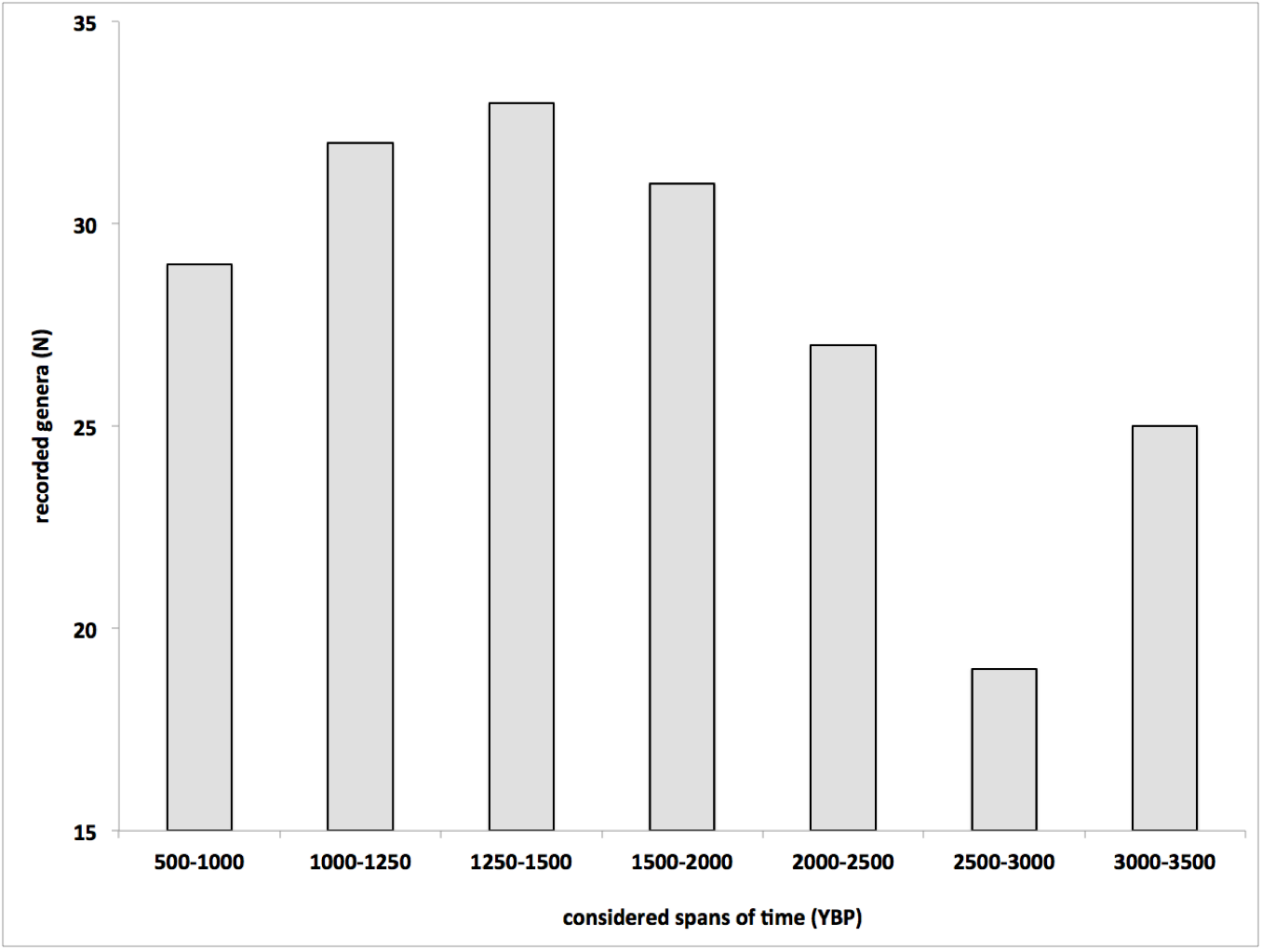
Number of sponge genera recorded in the considered periods. Note the sharp decreases corresponding to the temperature collapse at the end of the Bronze Age (3000–2500 YBP). After this period, the sponge diversity progressively increased during the Little Climatic Optimum (2500–1500 YBP) to decrease again during the Dark Age Cold Period (1500–1000 YBP).

The sponge diversity at generic level, evaluated at 500/250-year intervals, ranged between 19 and 33 genera, with a trend characterised by a sharp decreases from 3500–3000 to 3000–2500 YBP. After this period, the number of recorded genera regularly increased until 1500–1250 YBP and then progressively decreased until 1000–500 YBP (Fig 6).

**Fig 7:**
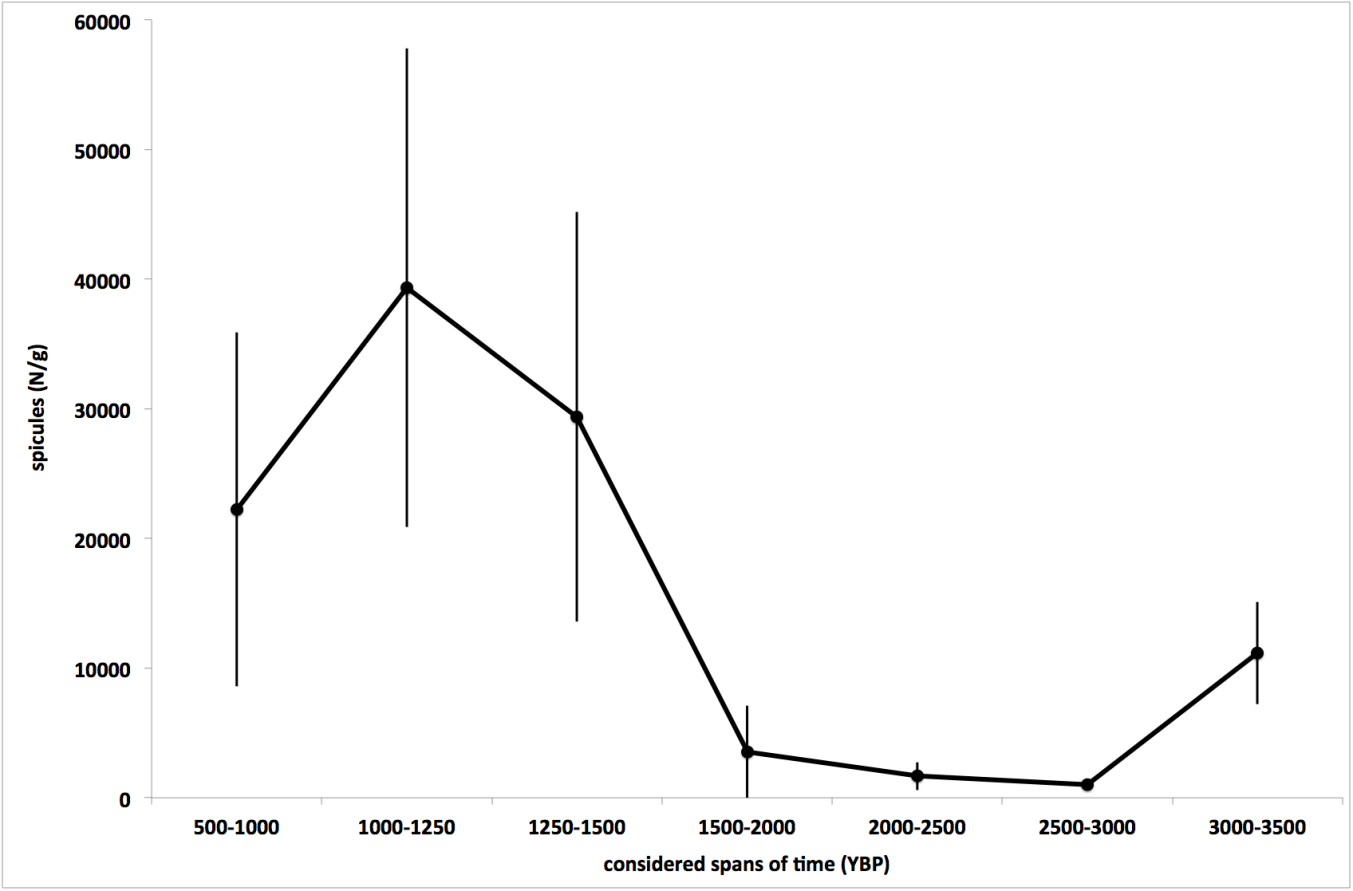
Average total spicule amount per sediment g (±SE) in each considered period.

From a quantitative point of view, it was observed that the total number of recorded spicules slightly decreased from 3500–3000 to 3000–2500 YBP and remained about constant until to 2000–1500 YBP. After this period, the value strongly increased in the period 1250–1000 YBP to decrease again in the most recent period (Fig 7).

The dominant spicules inside the bioherms were always the micrasters, belonging to some genera of the sub-order Astrophorina (probably *Jaspis*, *Geodia*, *Stelletta* and others). This quantity ranged from 75 to 87% of all the recorded spicules in all the considered periods.

## Discussion

The sponge fauna of the Bogliasco coralligenous build-ups was analysed over both a decennial and millennial spans of time. As already stated in similar habitats [22], the sponge assemblage showed a patchy distribution with significant differences in species richness among the considered build-ups. The comparison of the sponge diversity, recorded during the present survey, as well as that studied by Pansini and Pronzato [16] indicated a significant reduction of species richness (about 45%) in 40 years. The same analysis, conducted at generic level, indicated a loss of 20% of the genera present in the survey of 1973. This evidence is in agreement with the already known simplification of the superficial benthic communities of the Mediterranean Sea occurred mainly as a consequence of a number of mass mortality events that took place also in the Ligurian Sea since 1986, when sponges were the principal casualties [15, 23, 24, 25, 26, 27]. However, analysing the drop of the sponge diversity in this area, we cannot even rule out the impact of the date-mussels fishing conduced on the coralligenous build-ups until 1998, when this destructive activity was banned along the Italian coasts [28]. It was already stated the long time necessary for the recovering of the benthic communities deeply stressed by this fishing [29].

Both at specific and generic level, the massive/erect sponges, and in particular the Keratosa, were the most prone to change in the last decades, suggesting that the species living in the inner habitat of the bioherms were less affected by the variations of the environmental conditions or by the human pressures than those present on the substratum surface.

Nevertheless also some cavity dwelling species as *Triptolemma simplex* and *Timea* spp., that 40 years ago were frequent, disappeared in the Bogliasco area. These species were recently recorded, although at major depth, along the Portofino Promontory that is close to the studied site [11, 14]. This depth change is in agreement with the effects of the global warming which drove some littoral species to disappear from shallow waters and eventually to move down deeper, within their bathymetric range of distribution, a phenomenon already recorded for other shallow water taxonomic groups [30, 31, 32].

**Fig 8:**
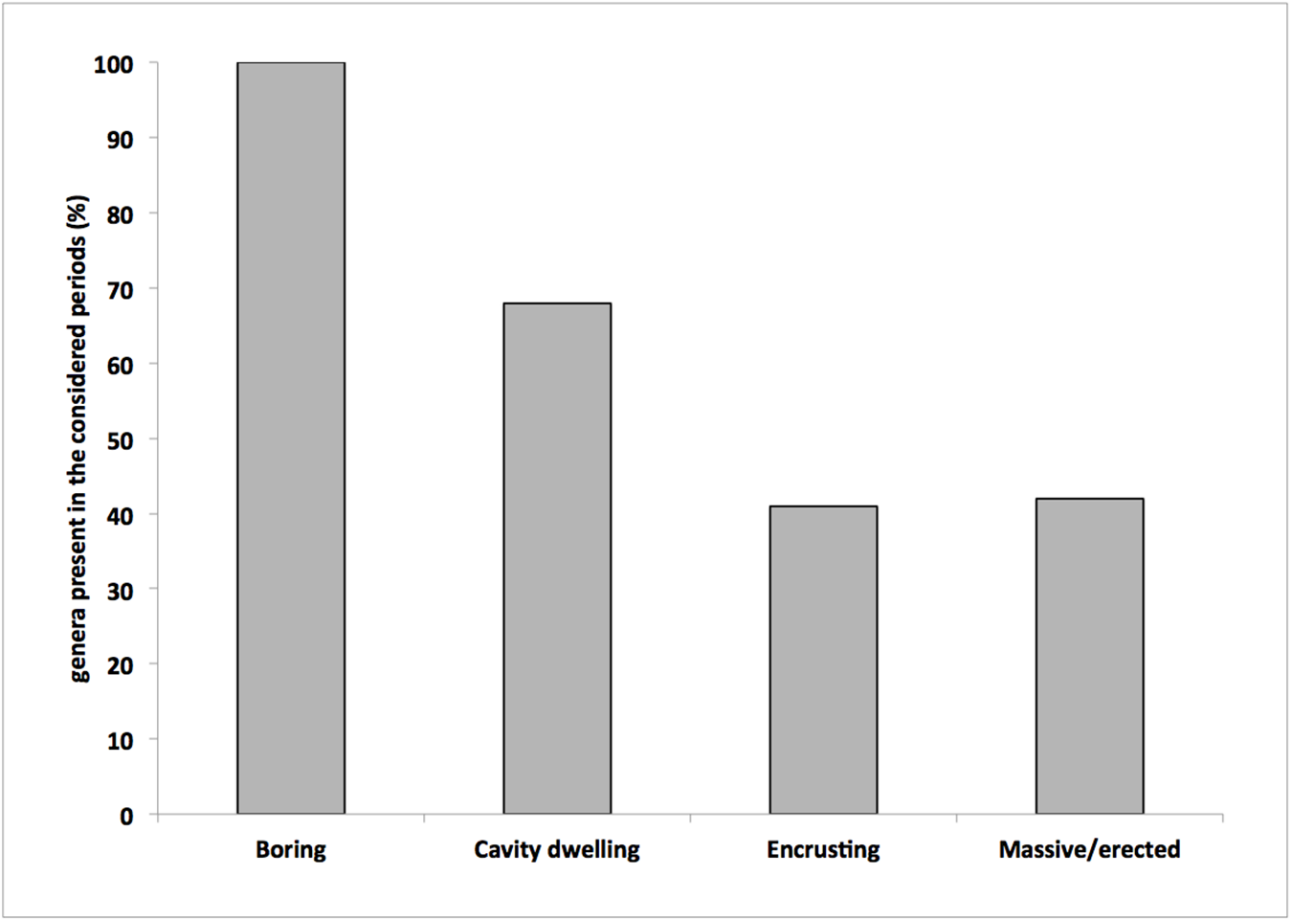
Percent of sponge genera recorded in all the considered periods according to the different growth patterns.

Through the study of millennial variations of the sponge assemblage, we have presently recorded 24 genera of the ancient-assemblages (60%), suggesting a remarkable stability across a 3500 yrs span of time. However, in terms of growth patterns the sponge diversity did not remain homogeneous during this long span of time. It showed a strong reduction of massive/erect species, while the cavity dwelling and boring ones decreased less (Fig 8). This evidence confirms, also on millennial span of time, the higher stability of the inner coralligenous habitat and underlines the strong attractiveness to sponge colonisation of this peculiar habitat both in terms of species diversity and biomass [11, 33].

We have also to consider that in 3500 years the Mediterranean Sea witnessed at least five different climatic periods: the Late Bronze Age Collapse, a sharp temperature decrease occurred about 3000–2500 yrs BP, the Little Climate Optimum, a gradual drying and warm period between the Greek Classical Period (5^th^ century BC) and the Late Roman Period (4^th^ century AD), the Dark Ages Cold Period, a second short and cooler period between 4^th^ and 9^th^ century, the Medieval Warm Period (MWP), which characterised the Europe between 10^th^ and 14^th^ century, and finally the Little Ice Age (LIA) that last from the 16^th^ to the 19^th^ centuries [34, 35, 36, 37, 38]. For example, the Medieval Warm Period favoured glacial melting which, ultimately, resulted in a salinity decrease and in a drop of 1–4°C in surface sea-water temperature [39], while the carbonate deposition seems to have had a halt. In fact, reduced air temperatures in spring and winter, or stochastic phenomena as floods, could have determined changes in seawater temperatures, salinity, turbidity and sediment regimes which, in turn, could have had relevant impact on the development of these bio-structures.

**Fig 9:**
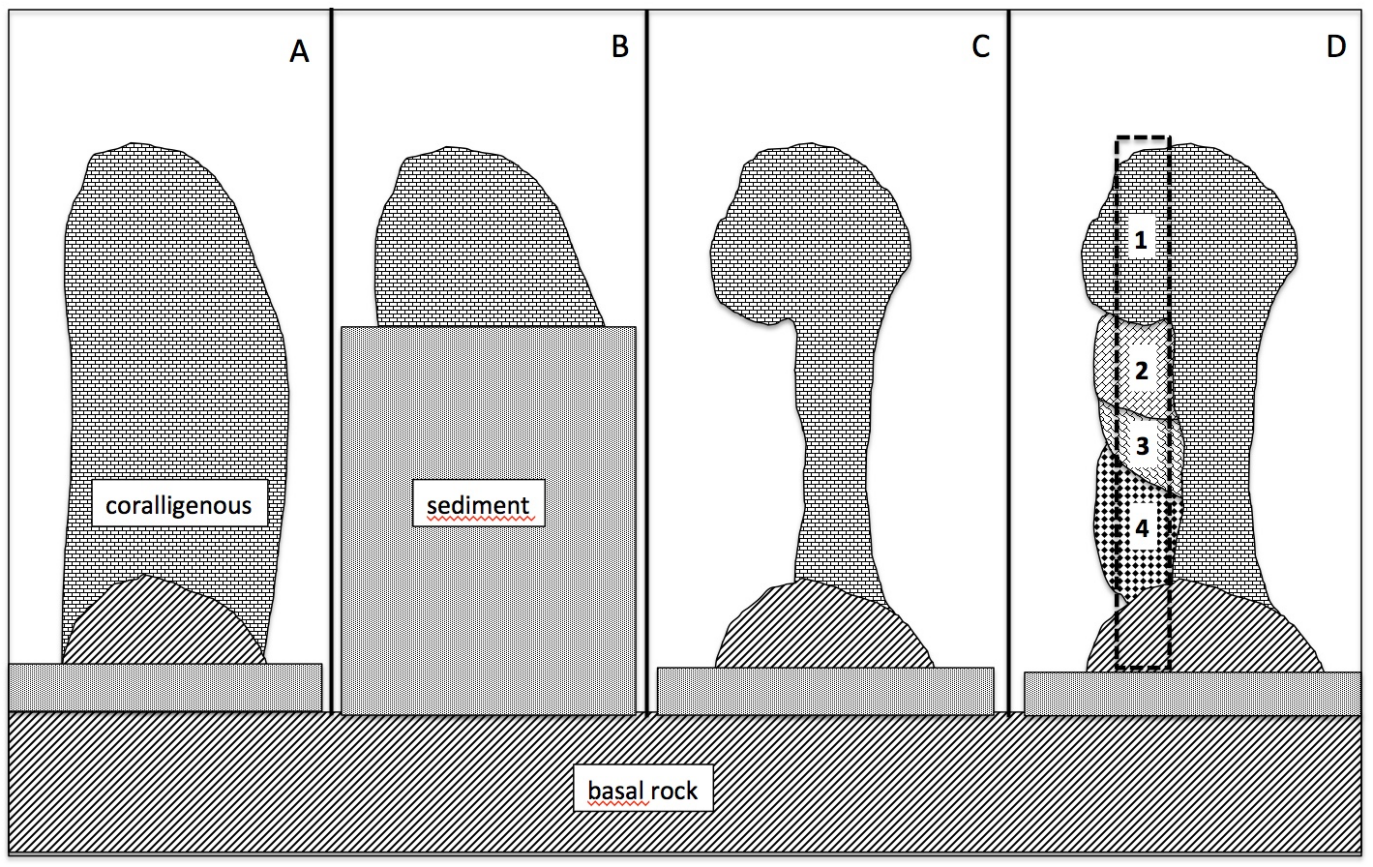
Hypothetical evolutionary scenario of the coralligenous accretions of Bogliasco. A) In a first phase the algal growth resulted in pillar-like bioherm. B) Periods of heavy floods could have increased the bottom sediments, partially or totally burying the pillars and killing the algal coverage. C) During the burying or after the removal of the sediments, a part of the structure could be prone to erosive processes, giving rise to mushroom-like structures. D) In following phases, the coralline algae could grow again in sciaphilous microhabitats, determining the irregular temporal layering of the structure (the number from 1 to 4 indicated different sheets of deposition from the oldest to the youngest). In this situation, in a core sample (dotted rectangle), younger sheets can be overlapped by older ones.

This kind of phenomena can be at the base of the incoherent age determination of the coralligenous bioconstructions of Bogliasco. This puzzling situation, with older layers overlapping younger ones, could be related to favourable phases for carbonate deposition, alternate with partial destruction phases imputable, for example, to intense mud deposition after episodes of important floods. Fig 9 shows a hypothetical evolutionary scenario characterised by a first phase of algal growth on the rocky substrata resulting in pillar-like bioherm (Fig 9A), similar to those recorded from several regions of the Southern Mediterranean [8]. Periods of heavy floods could have increased the bottom sediments, partially or totally burying the pillars and killing the algal coverage (Fig 9B). During the burying or after the removal of the sediments, a part of the structure could be prone to erosive processes, giving rise to mushroom-like structures (Fig 9C). In following phases, the coralline algae could grow again in sciaphilous microhabitats, determining the irregular temporal layering of the structure (Fig 9D). In this situation, in a core sample, younger sheets can be overlapped by older ones.

**Fig 10:**
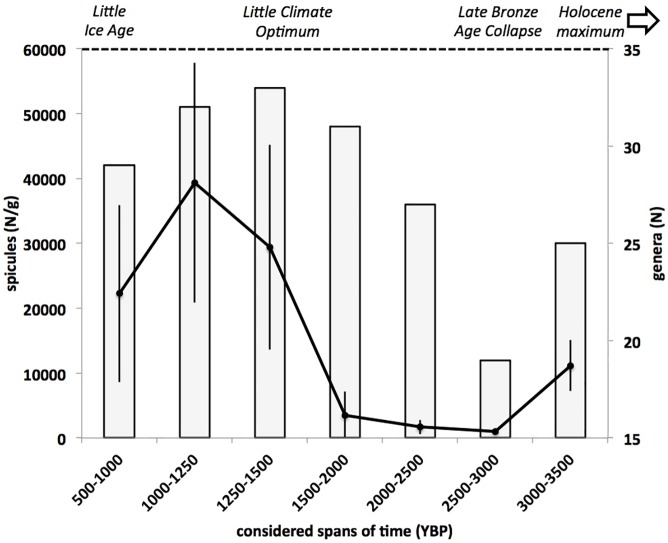
Trend of sponge diversity evaluated as number of genera present in each period (grey bars) compared with the trend of sponge abundance evaluated as average amount of spicules per sediment g present in the same periods.

The temperature variations at millennial scale seemed to affect also the sponge richness: in fact, the number of genera observed in the bioherms decreased contemporaneously to the Late Bronze Age Collapse, to progressively increase during the Little Climate Optimum and to decrease again in the Dark Ages Cold Period. This datum is in good agreement with the sponge abundance (evaluated as average number of spicules) (Fig 10), suggesting that the warmer periods favoured the proliferation of the sponge assemblages.

Also recent studies have put in evidence different responses in facing the global warming. In the Aegean Isle of Kos, for example, an increase, by one order of magnitude, of the overall abundance of the horny sponge *Ircinia retidermata* was estimated [40]. On the contrary, in a pre-coralligenous assemblage of the Portofino Marine Protected Area, sponges remained almost constant in terms of total coverage over a span of time of 25 years; red encrusting sponges (mainly *Crambe crambe* and *Spirastrella cunctatrix*) were always the most abundant, but the massive/erect ones suffered the major losses [11, 41].

It is remarkable that the comparison of the genera recorded in ancient assemblages and modern ones showed that the amounts of genera with cavity dwelling and encrusting habits remained quite constant, maintaining a comparable diversity along the entire development of the bioherms. On the contrary, the boring genera shifted from four in ancient assemblages, to only one. This evidence is in agreement with the shift of ancient boring communities dominated by the genera *Alectona*, *Thoosa*, *Dotona* to modern communities mainly characterised by species belonging to the genus *Cliona*, as already recorded in coral reefs [42]. More caution is necessary in the evaluation of the increasing of massive/erect sponge genera in recent communities. In fact, a number of these sponges have no siliceous spicules and it is reasonable to expect that the spicules of massive species living on the build-up surface have less probability to be trapped into it.

The coralligenous communities are habitats of European Community interest (Flora Fauna Habitat Directive 92/43/EC, 1170–1114 Code: reefs) due to their high species richness and their important role in the balance of the carbonates at sea [2, 43, 44]. Moreover, they are potential indicators in monitoring the environmental quality of coastal waters according to the Marine Strategy Framework Directive (2008/56/EC). Unfortunately, these habitats are very vulnerable and their structures and biodiversity threatened not only by the changing climate conditions and temperature anomalies [45], but also by pollution, excessive sedimentation, turbidity, biological invasions, fishing and diving activities, and by the synergistic effects of all these stressors [26, 46, 47]. Therefore these marine ecosystems have a high conservation value and it is required to maintain and restore their proper functioning to enable their preservation for future generations.

The concerns raised in recent years towards the depletion of these habitats seem to be confirmed by the results of this study. In fact, the sponge fauna of the upper coralligenous, one of the most representative taxa of these habitats [14], has suffered in recent years a strong decrease in quali- and quantitative terms, showing, in the Ligurian Sea, a depletion which appears to have never been recorded before. The taxonomic analysis of siliceous sponge spicules, a powerful tool for a tentative reconstruction of ancient sponge assemblages [48, 49, 50], indicated that the sponge genera present in the coralligenous habitats remained enough stable in the last 3500 years [11].

## Supporting information

S1 Table Modern sponge species recorded in the 15 scraped standard areas and relative percent presence. (DOCX)

S2 Table Presence of spicules referred to different genera or supergeneric taxa in core sample 1. (DOCX)

S3 Table Presence of spicules referred to different genera or supergeneric taxa in core sample 2. (DOCX)

S4 Table Presence of spicules referred to different genera or supergeneric taxa in core sample 3. (DOCX)

S5 Table Presence of spicules referred to different genera or supergeneric taxa in core sample 4. (DOCX)

S6 Table Presence of spicules referred to different genera or supergeneric taxa in core sample 5. (DOCX)

## References

[1] Bavestrello G, Bianchi CN, Calcinai B, Cattaneo-Vietti R, Cerrano C, Morri C et al (2000) Bio-mineralogy as a structuring factor for marine epibenthic communities. Mar Ecol Progr Ser 193: 241–249.

[2] Ballesteros E (2006) Mediterranean coralligenous assemblages: a synthesis of present knowledge. Oceanog Mar Biol: An Rev 44: 123–195.

[3] Laborel J (1961) Le concrétionnement algal “coralligène” et son importance géomorphologique en Méditerranée. R Trav Stn Mar Endoume 23: 37–60.

[4] Laubier L (1966) Le coralligène des Albères: monographie biocénotique. Ann Inst Océanogr Paris 43: 139–316.

[5] Sartoretto S, Verlaque M, Laborel J (1996) Age of settlement and accumulation rate of submarine “coralligène” (-10 to -60 m) of the north-western Mediterranean Sea; relation to Holocene rise in sea level. Mar Geol 130: 317–331.

[6] Hong JS (1980) Etude faunistique d’un fond de concrétionnement de type coralligène soumis à un gradient de pollution en Méditerranée nord-occidentale (Golfe de Fos). Thèse Dr Univ Aix- Marseille II: 1–268.

[7] Ros J, Romero J, Ballesteros E, Gili J (1985) The circalittoral hard bottom communities: the coralligenous In: Margalef R ed. Western Mediterranean. Pergamon Press, Oxford pp 263–273.

[8] Di Geronimo I, Di Geronimo R, Rosso A, Sanfilippo R (2002) Structural and taphonomic analysis of a columnar coralline algal build-up from SE Sicily. Geobios 35: 86–95.

[9] Garrabou J, Ballesteros E (2000) Growth of *Mesophyllum alternans* and *Lithophyllum frondosum* (Corallinales, Rhodophyta) in the northwestern Mediterranean. Eur J Phycol 35: 1–10.

[10] Teixidó N, Garrabou J, Harmelin JG (2011) Low dynamics, high longevity and persistence of sessile structural species dwelling on Mediterranean coralligenous outcrops. PLoS One 6. 10.1371/journal.pone.0023744 PMC3161055 21887308

[11] Bertolino M, Calcinai B, Cattaneo-Vietti R, Cerrano C, Lafratta A, Pansini M et al (2014) Stability of the sponge assemblage of the Mediterranean coralligenous along a millennial span of time. Mar Ecol 35: 149–158.

[12] Sarà M, Pulitzer-Finali G (1970) Nuove vedute sulla classificazione del coralligeno. Pubbl Staz Zool Napoli 38 (suppl.): 174–179.

[13] Got H, Laubier L (1968) Prospection sismiques au large des Albères: nature du substrat originel des fonds coralligènes. Vie et Milieu 19: 9–16.

[14] Bertolino M, Cerrano C, Bavestrello G, Carella M, Pansini M, Calcinai B (2013) Diversity of Porifera in the Mediterranean coralligenous accretions, with description of a new species. ZooKeys 336: 1–37. 10.3897/zookeys.336.5139 PMC3800777 24146570

[15] Cerrano C, Bavestrello G, Bianchi CN, Cattaneo-Vietti R, Bava S, Morganti C et al (2000) A catastrophic mass-mortality episode of gorgonians and other organisms in the Ligurian Sea (North-western Mediterranean), summer 1999. Ecol Lett 3: 284–293.

[16] Pansini M, Pronzato R (1973) Il coralligeno di Bogliasco ed il suo popolamento di poriferi. Boll Mus Ist Biol Univ Genova 41: 5–34.

[17] Calcagnile L, Quarta G, D’Elia M (2005) High resolution accelerator-based mass spectrometry: precision, accuracy and background. Applied Radiation and Isotopes 62: 623–626. doi: 10.1016/j.apradiso.2004.08.047 15701419 10.1016/j.apradiso.2004.08.047

[18] D’Elia M, Calcagnile L, Quarta G, Rizzo A, Sanapo C (2004) Sample preparation and blank values at the AMS radiocarbon facility of the University of Lecce. Nucl Instr Met Phys Res B 223–224: 278–283.

[19] Reimer PJ, Bard E, Bayliss Alex, Beck JW, Blackwell PG, Bronk Ramsey C et al (2013) IntCal13 and Marine13 Radiocarbon Age Calibration Curves 0–50,000 Years cal BP. Radiocarbon 55: 1869–1887.

[20] Reimer PJ, McCormac FG (2002) Marine radiocarbon reservoir corrections for the Mediterranean and Aegean sea. Radiocarbon 44: 159.

[21] Santos GG, Pinheiro U, Hajdu E, Van Soest R (2016) New genus and species of Heteroxyidae from Brazil (Axinellida: Demospongiae: Porifera), with a revised identification key for the family. Zootaxa 4158 (1): 105–116. doi: 10.11646/zootaxa.4158.1.6 27615873 10.11646/zootaxa.4158.1.6

[22] Garrabou J, Ballesteros E, Zabala M (2002) Structure and dynamics of north-western Mediterranean rocky benthic communities along a depth gradient. Estuarine, Coastal and Shelf Science 55(3): 493–508.

[23] Gaino E, Pronzato R, Corriero G, Buffa P (1992) Mortality of commercial sponges: incidence in two Mediterranean areas. It J Zool 59 (1): 79–85.

[24] Coma R, Ribes M, Serrano E, Jimeneza E, Salat J, Pascual J (2009) Global warming-enhanced stratification and mass mortality events in the Mediterranean. Proc Nat Acad Sci 106: 6176–6181. doi: 10.1073/pnas.0805801106 19332777 10.1073/pnas.0805801106 PMC2669359

[25] Garrabou J, Coma R, Bensoussan N, Bally M, Chevaldonne P, Cigliano M et al (2009) Mass mortality in Northwestern Mediterranean rocky benthic communities: effects of the 2003 heat wave. Global Change Biology 15: 1090–1103.

[26] Maldonado M, Sanchez-Tocino L, Navarro C (2010) Recurrent disease outbreaks in corneous demosponges of the genus *Ircinia*: epidemic incidence and defence mechanisms. Mar Biol 157: 1577–1590.

[27] Cebrian E, Uriz MJ, Garrabou J, Ballesteros E (2011) Sponge mass mortalities in a warming Mediterranean Sea: are cyanobacteria-harboring species worse off? PLoS One, 6, e20211 doi: 10.1371/journal.pone.0020211 21673798 10.1371/journal.pone.0020211 PMC3105983

[28] Fraschetti S, Bianchi CN, Terlizzi A, Fanelli G, Morri C Boero F (2001) Spatial variability and human disturbance in shallow subtidal hard substrate assemblages: a regional approach. Mar Ecol Progr Ser 212: 1–12.

[29] Fanelli G, Piraino S., Belmonte G, Geraci S, Boero F (1994) Human predation along Apulian rocky coasts (SE Italy): desertification caused by *Lithophaga lithophaga* (Mollusca) fisheries. Mar Ecol Progr Ser 110 (1): 1–8.

[30] Cerrano C, Totti C, Sponga F, Bavestrello G (2006) Summer disease in *Parazoanthus axinellae* (Schmidt, 1862) (Cnidaria, Zoanthidea). It J Zool 73(4): 355–361.

[31] Puce S, Bavestrello G, Di Camillo CG, Boero F (2009) Long-term changes in hydroid (Cnidaria, Hydrozoa) assemblages: effect of Mediterranean warming? Mar Ecol 30 (3): 313–326.

[32] Burrows MT, Schoeman DS, Buckley LB, Moore P, Poloczanska ES, Brander KM et al (2011) The pace of shifting climate in marine and terrestrial ecosystems. Science 334 (6056): 652–655. doi: 10.1126/science.1210288 22053045 10.1126/science.1210288

[33] Calcinai B, Bertolino M, Bavestrello G, Montori S, Mori M, Pica D et al (2015) Comparison between the sponge fauna living outside and inside the coralligenous bioconstruction. A quantitative approach. Medit Mar Sc 16(2): 413–418.

[34] Dansgaard W, Johnsen SJ, Clausen HB, Dahl-Jensen D, Gundestrup N, Hammer CU et al (1984) North Atlantic Climatic Oscillations Revealed by Deep Greenland Ice Cores In: Hansen JE, Takahashi T eds. Climate Processes and Climate Sensitivity. American Geophysical Union. John Wiley & Sons Ltd.

[35] Reale O, Dirmeyer P (2000) Modeling the effects of vegetation on Mediterranean climate during the Roman Classical Period: Part I: Climate history and model sensitivity. Global and Planetary Change 25(3): 163–184.

[36] Mann ME (2002) Little ice age. Encyclopaedia of global environmental change 1: 504–509.

[37] Sicre MA, Jacob J, Ezat U, Rousse S, Kissel C, You P et al (2008) Decadal variability of sea surface temperatures off North Iceland over the last 2000 years. Earth and Planetary Science Letters 268 (1): 137–142.

[38] Drake BL (2012) The influence of climatic change on the Late Bronze Age Collapse and the Greek Dark Ages. J Arch Sc 39: 1862–1870.

[39] Taricco C, Ghil M, Alessio S, Vivaldo G (2009) Two millennia of climate variability in the Central Mediterranean. Clim Past 5: 171–181.

[40] Bianchi CN, Corsini-Foka M, Morri C, Zenetos A (2014) Thirty years after: dramatic change in the coastal marine ecosystems of Kos Island (Greece), 1981–2013. Medit Mar Sc 15 (3): 482–497.

[41] Betti F, Bavestrello G, Bo M, Asnaghi V, Chiantore M, Bava S et al (2017) Over 10 years of variation in Mediterranean reef benthic communities. Mar Ecol (in press).

[42] Wiedenmayer F (1994) Contributions to the knowledge of post-Palaeozoic neritic and archibenthal sponges (Porifera): the stratigraphic record, ecology, and global distribution of intermediate and higher taxa. Birkhäuser Publ.

[43] Martin S, Gattuso JP (2009) Response of Mediterranean coralline algae to ocean acidification and elevated temperature. Global Change Biology 15 (8): 2089–2100.

[44] Boudouresque CF, Blanfuné A, Harmelin-Vivien M, Personnic S, Ruitton S, Thibaut T et al (2016) Where Seaweed Forests Meet Animal Forests: the Examples of Macroalgae in Coral Reefs and the Mediterranean Coralligenous Ecosystem In: Rossi S, Bramanti L, Gori A, Orejas Saco del Valle C eds. Marine Animal Forests. The Ecology of Benthic Biodiversity Hotspots. Springer Verlag pp 1–28.

[45] Cerrano C, Bavestrello G, Bianchi CN, Calcinai B, Cattaneo-Vietti R, Morri C et al (2001) The role of sponge bioerosion in the Mediterranean coralligenous accretion In: Faranda FM, Guglielmo L, Spezie G eds. Mediterranean Ecosystems: structure and processes. Springer Verlag, Italy pp 235–240.

[46] Piazzi L, Gennaro P, Balata D (2012) Threats to macroalgal coralligenous assemblages in the Mediterranean Sea. Mar Poll Bull 64 (12): 2623–2629. 10.1016/j.marpolbul.2012.07.027 22863350

[47] Teixidó N, Casas E, Cebrián E, Linares C, Garrabou J (2013) Impacts on coralligenous outcrop biodiversity of a dramatic coastal storm. PLoS One 8(1): e53742 doi: 10.1371/journal.pone.0053742 23326496 10.1371/journal.pone.0053742 PMC3542355

[48] Łukowiak M (2016) Fossil and modern sponge fauna of southern Australia and adjacent regions compared: interpretation, evolutionary and biogeographic significance of the late Eocene ‘soft’sponges. Contr Zool 85(1): 13–35.

[49] Łukowiak M, Pisera A, O'Dea A (2013) Do spicules in sediments reflect the living sponge community? A test in a Caribbean shallow-water lagoon. Palaios 28(6): 373–385.

[50] Frisone V, Pisera A, Hajdu E, Preto N, Zorzi F, Zorzin R (2014) Isolated spicules of Demospongiae from Mt. Duello (Eocene, Lessini Mts., Northern Italy): preservation, taxonomy, and depositional environment. Facies 60(4): 883–904.

