# Supplementary material for: The dynamics of a Mediterranean coralligenous sponge assemblage at decennial and millennial temporal scales
Source: PLoS One. 2017 May 22;12(5):e0177945. doi: 10.1371/journal.pone.0177945 (PMC5439943; doi:10.1371/journal.pone.0177945)
Supplement: S1 Table — (DOCX) [file pone.0177945.s001.docx]

Table S1 – Modern sponge species recorded in the 15 scraped standard areas and relative percent presence.

| **Sponge species** | **S1** | **S22** | **S3** | **S4** | **S5** | **S6** | **S7** | **S8** | **S9** | **S10** | **S11** | **S12** | **S13** | **S14** | **S15** | **% presence** |
| --- | --- | --- | --- | --- | --- | --- | --- | --- | --- | --- | --- | --- | --- | --- | --- | --- |
| *Agelas oroides* Schmidt, 1864 |  |  | X |  |  | X |  |  |  |  |  | X |  |  |  | **20** |
| *Axinella damicornis* (Esper, 1794) |  |  |  |  |  |  |  | X |  |  | X |  |  |  |  | **13** |
| *Eurypon cinctum* Sarà, 1960 |  |  |  |  |  |  |  |  |  |  |  |  |  |  | X | **7** |
| *Eurypon clavatum* (Bowerbank, 1866) |  |  |  |  |  |  |  |  |  |  |  | X |  |  |  | **7** |
| *Eurypon major* Sarà & Siribelli, 1960 |  |  | X |  |  |  |  |  | X |  |  |  |  |  |  | **13** |
| *Eurypon vescicularis* Sarà & Siribelli, 1960 | X |  |  |  |  |  |  |  |  |  | X |  |  |  |  | **13** |
| *Eurypon* sp. |  |  | X |  |  |  |  |  |  |  |  |  |  |  |  | **7** |
| *Bubaris vermiculata* (Bowerbank, 1866) |  |  | X |  |  |  |  |  |  |  |  |  |  |  |  | **7** |
| *Acanthella acuta* Schmidt, 1862 | X |  |  |  |  |  |  |  |  |  | X | X |  |  |  | **20** |
| *Dictyonella incisa* (Schmidt,1880) |  |  |  |  |  |  |  |  |  |  | X | X |  |  |  | **13** |
| *Cliona celata* Grant, 1826 |  |  |  |  |  |  |  |  |  |  | X |  |  |  |  | **7** |
| *Cliona janitrix* Topsent, 1932 |  |  |  |  |  |  |  |  |  |  |  |  |  |  | X | **7** |
| *Cliona schmidtii* (Ridley, 1881) |  |  | X |  |  |  | X |  |  |  |  |  |  |  |  | **13** |
| *Cliona viridis* Schmidt, 1862 |  | X |  | X |  |  | X |  |  |  |  |  |  | X |  | **27** |
| *Spirastrella cunctatrix* Schmidt, 1868 |  |  |  |  |  |  |  |  |  |  | X | X |  |  |  | **13** |
| *Haliclona (Gellius) lacazei* (Topsent, 1893) |  |  |  |  |  |  |  |  |  |  |  |  |  | X | X | **13** |
| *Haliclona (Reniera)* cf. *mediterranea* Griessinger, 1971 |  |  |  |  |  |  | X | X |  |  |  |  |  |  |  | **13** |
| *Haliclona (Reniera)* sp. |  | X |  |  |  |  |  |  |  |  |  | X |  |  | X | **20** |
| *Haliclona* (*Soestella*) *valliculata* (Griessinger, 1971) |  |  |  |  |  |  |  |  |  |  |  |  |  | X | X | **13** |
| *Petrosia (Petrosi a) ficiformis* (Poiret,1798) | X |  | X |  |  |  |  |  |  | X | X |  |  |  |  | **27** |
| *Oceanapia* sp. |  |  |  |  |  |  |  |  |  |  |  |  |  | X |  | **7** |
| *Batzella inops* (Topsent, 1891) |  |  |  |  |  |  |  |  | X |  |  |  |  |  |  | **7** |
| *Crambe crambe* (Schmidt,1862) | X | X |  | X | X | X | X | X |  | X | X | X |  |  | X | **73** |
| *Hymedesmia* (*Hymedesmia*) *baculifera* (Topsent, 1901) |  |  |  |  |  |  |  |  |  |  |  |  |  |  | X | **7** |
| *Hymedesmia (Hymedesmia)* cf. *gracilisigma* Topsent, 1928 |  |  |  |  |  |  |  |  |  |  |  | X |  |  |  | **7** |
| *Hymedesmia (Hymedesmia)* sp. |  |  |  |  |  |  | X |  |  |  |  |  |  |  |  | **7** |
| *Phorbas fictitius* Bowerbank, 1866 |  | X |  |  |  |  | X |  |  |  |  |  | X |  |  | **20** |
| *Phorbas tenacior* (Topsent, 1925) |  | X |  |  | X |  | X | X | X |  |  | X |  |  |  | **40** |
| *Mycale (Mycale) massa* (Schmidt, 1862) |  |  |  |  |  |  |  |  |  |  | X |  |  |  | X | **13** |
| *Myxilla* (*Myxilla*) *rosacea* (Lieberkühn, 1859) |  |  |  |  |  |  |  |  |  |  | X |  |  |  |  | **7** |
| *Polymastia mamillaris* (Müller, 1806) | X |  |  |  |  |  |  |  |  |  |  |  |  |  |  | **7** |
| *Halichondria (Halichondria) genetrix* (Schmidt, 1870) |  |  |  | X | X |  |  |  |  |  |  | X |  |  |  | **20** |
| *Halichondria (Halichondria) panicea* (Pallas, 1766) |  |  |  |  |  |  |  |  |  |  |  |  | X |  |  | **7** |
| *Halichondria* sp. |  |  |  |  |  |  |  |  |  |  |  | X |  | X | X | **20** |
| Hymeniacidon perlevis (Montagu, 1818) |  |  |  |  |  |  |  | X |  |  |  |  |  |  |  | **7** |
| Aaptos aaptos (Schmidt,1864) |  | X | X |  |  |  |  |  |  |  | X |  |  |  | X | **27** |
| *Terpios gelatinisa* (Bowerbank, 1866) |  |  |  |  |  |  |  |  |  |  |  | X |  |  |  | **7** |
| *Dercitus (Stoeba) plicatus*  (Schmidt, 1868) | X | X | X |  | X | X | X | X | X |  |  |  | X | X | X | **73** |
| *Jaspis incrustans* (Topsent, 1890) | X | X |  |  |  |  |  | X |  |  |  |  |  | X | X | **33** |
| *Jaspis johnstonii* (Schmidt,1862) | X | X |  |  | X |  | X | X | X | X |  | X |  | X | X | **67** |
| *Stelletta lactea* Carter, 1871 |  |  |  |  |  |  |  |  |  |  |  |  |  | X |  | **7** |
| *Erylus discophorus* (Schmidt, 1862) |  |  |  |  |  | X |  |  | X |  |  |  |  |  | X | **20** |
| *Ircinia variabilis* (Schmidt, 1862) |  |  |  |  |  |  |  |  |  |  |  | X |  |  |  | **7** |
| *Sarcotragus spinosulus* Schmidt, 1862 | X | X |  |  |  |  |  |  |  |  |  |  |  |  |  | **13** |
| *Spongia (Spongia) virgultosa* (Schmidt, 1868) |  |  |  | X |  |  |  |  |  |  | X |  |  | X |  | **20** |
| *Fasciospongia cavernosa* (Schmidt, 1862) |  |  |  |  |  |  |  |  |  | X |  |  |  |  |  | **7** |
| *Chondrosia reniformis* Nardo,1847 | X | X |  |  |  |  | X |  | X |  |  | X |  |  |  | **33** |
| *Plakina trilopha* Schulze, 1880 |  |  | X |  |  |  |  |  |  |  |  |  |  |  | X | **13** |
| *Oscarella lobularis* (Schmidt, 1862) |  |  |  |  |  |  |  |  |  |  |  |  | X |  |  | **7** |
| **Total species recorded in the scraped area** | **10** | **11** | **9** | **4** | **5** | **4** | **10** | **8** | **7** | **4** | **12** | **15** | **4** | **10** | **15** |  |
