# Supplementary material for: The dynamics of a Mediterranean coralligenous sponge assemblage at decennial and millennial temporal scales
Source: PLoS One. 2017 May 22;12(5):e0177945. doi: 10.1371/journal.pone.0177945 (PMC5439943; doi:10.1371/journal.pone.0177945)
Supplement: S6 Table — (DOCX) [file pone.0177945.s006.docx]

Table S6 – Presence of spicules referred to different genera or supergeneric taxa in core sample 5

| **Sponge taxa** | **Growth pattern** | **Distance from the basal rock (cm)/ Calibrated radiocarbon ages (YBP)** | | | | |
| --- | --- | --- | --- | --- | --- | --- |
|  |  | **0-3** | **3-6** | **6-9** | **9-12** | **12-15** |
|  |  | **1974 ± 65** | **3442 ± 55** | **2422 ± 80** | **871 ± 55** | **3104 ± 75** |
| **Subclass** Heteroscleromorpha |  | X | X |  |  |  |
| *Agelas* | **ME** |  |  |  |  |  |
| *Alveospongia* | **ME** |  |  |  | X | X |
| **Family** Raspailiidae |  |  |  |  |  | X |
| *Eurypon* | **En** |  | X | X | X | X |
| *Rhabderemia* | **ME** | X | X | X | X | X |
| *Bubaris* | **En** |  |  |  |  |  |
| **Family** Dictyonellidae |  |  |  |  |  |  |
| *Acanthella* | **ME** |  |  | X | X |  |
| **Family** Clionaidae |  | X | X | X | X | X |
| *Dotona* | **Cd** | X | X | X | X | X |
| *Cliona* | **Br** | X | X | X | X | X |
| *Spiroxya* | **Br** | X | X | X | X | X |
| *Placospongia* | **En** |  | X |  |  |  |
| **Family** Spirastrellidae |  | X | X |  |  |  |
| *Diplastrella* | **En** |  | X | X | X |  |
| **Family** Chalinidae |  | X | X | X | X | X |
| *Dendroxea* | **Cd** |  |  |  |  |  |
| *Haliclona (Gellius)* |  |  |  |  |  | X |
| *Petrosia (Petrosia)* | **ME** |  |  |  |  |  |
| **Order** Poecilosclerida |  | X | X | X | X | X |
| *Acarnus* | **Cd** | X | X | X |  | X |
| *Batzella* | **En** |  |  |  |  |  |
| *Forcepia* | **En** |  |  |  |  |  |
| *Crella* | **En** |  |  |  | X | X |
| **Family** Hymedesmiidae |  |  |  |  |  |  |
| *Clathria* | **En** |  |  |  |  |  |
| *Antho* | **En** |  | X | X | X | X |
| *Mycale* | **En** |  |  |  | X |  |
| *Myxilla* | **En** |  |  |  |  |  |
| **Family** Halichondriidae |  |  |  |  |  |  |
| **Family** Suberitidae |  |  |  |  |  |  |
| *Aaptos* | **Cd** |  | X | X |  |  |
| *Protosuberites* | **En** | X | X | X |  |  |
| *Tethya* | **ME** | X | X |  |  |  |
| *Timea* | **Cd** | X | X | X | X | X |
| **Family** Ancorinidae |  |  |  |  |  |  |
| *Dercitus (Stoeba)* | **Cd** | X | X | X | X | X |
| *Jaspis* | **Cd** | X | X | X | X | X |
| *Stelletta* | **Cd** |  |  |  |  |  |
| *Erylus* | **Cd** | X | X | X | X | X |
| *Geodia* | **Cd** | X | X | X | X | X |
| *Pachastrella* | **Cd** |  |  |  |  |  |
| *Triptolemma* | **Cd** |  |  |  |  |  |
| *Alectona* | **Br** |  | X |  |  |  |
| *Thoosa* | **Br** |  | X | X | X |  |
| *Samus* | **Cd** | X | X |  |  |  |
| *Chondrilla* | **ME** |  |  |  |  |  |
| **Family** Plakinidae |  | X | X | X | X | X |
| *Corticium* | **ME** |  |  |  |  |  |
| *Plakina* | **En** | X | X |  |  | X |
| *Plakortis* | **En** |  |  |  |  |  |
